# Physical Activity, Healthy Behavior and Its Motivational Correlates: Exploring the Spillover Effect through Stages of Change

**DOI:** 10.3390/ijerph19106161

**Published:** 2022-05-19

**Authors:** María Marentes-Castillo, Isabel Castillo, Inés Tomás, Octavio Alvarez

**Affiliations:** 1 Department of Social Psychology, University of Valencia, 46010 Valencia, Spain; maria.marentes@ext.uv.es (M.M.-C.); isabel.castillo@uv.es (I.C.); octavio.alvarez@uv.es (O.A.); 2 National Council for Science and Technology (CONACyT), Mexico City 03940, Mexico; 3 Department of Methodology of the Behavioral Sciences, University of Valencia, 46010 Valencia, Spain

**Keywords:** physical activity, weight control behaviors, stages of change, motivation

## Abstract

On the basis of the spillover or transfer effect and the transtheoretical model of change, this study assessed the association between amount of physical activity, healthy and unhealthy weight control behaviors, and motivational types, as well as their variability across stages of change. A total of 1219 randomly selected Mexican adults from 18 to 65 years old, representative of the city of Monterrey (México), participated in the study. Correlation analyses, differences by gender, and multivariate analyses of variance, controlling for age, were performed. We found that in the maintenance stage, there is higher frequency of physical activity more healthy weight control behaviors, as well as higher autonomous motivation. In the contemplation stage, there is less physical activity, a higher frequency of unhealthy weight control behaviors, higher controlled motivation, and amotivation. Relationships were found between the healthy behaviors studied and the interaction dynamics observed across the stages of change, highlighting the key role of the contemplation and maintenance stages in weight control change. Physical activity as a targeted intervention objective could be a gateway to healthier weight control behavior, as well as higher autonomous motivation.

## 1. Introduction

Physical activity and healthy behaviors are often associated with each other, and the same thing is true for risk behaviors. On the one hand, the initiation of one healthy behavior, such as physical activity, may lead to the initiation of another healthy behavior, such as weight control, and vice versa. On the other hand, a risky behavior such as smoking may lead to engaging in other risky behaviors, such as physical inactivity or alcoholism [1,2]. This association between healthy behaviors, and between unhealthy behaviors, or the transfer effect, has been called spillover [3,4,5]. Spillover refers to the fact that improvement in one healthy behavior leads to improvement in other healthy behaviors, as well as improvements in psychological factors such as self-efficacy, self-image, and coping [6].

The importance of studying the spillover effect lies in the search for the gateway from one healthy behavior to others, and although the results found have been inconsistent [5], studying this phenomenon has strong implications for the type of intervention proposed to promote healthy behavior, especially in increasing physical activity [1,2,7]. Physical activity has traditionally been considered a gateway behavior into other healthy behaviors and a determinant in the maintenance of a healthy weight [3,4,8].

The study of spillover and its implications for health improvement through the promotion of healthy lifestyle behaviors may be of great interest in implementing strategies of change towards these behaviors. Interventions to promote healthy behaviors that have tried to test the spillover effect have focused mainly on the practice of physical activity; behaviors related to food and/or diet; and, more recently, motivational variables, such as autonomy support from authority figures [4]. Thus, in the interventions during a one-year program for the promotion of physical activity, there were changes in eating-related behaviors, such as decreases in daily calorie, fat, and protein intake. However, these changes were not maintained after the intervention [9,10].

In addition, programs aimed at changing dietary habits through one-year intervention programs showed evidence of increased physical exercise [5,11], other diet-related behaviors such as improved weight and adiposity [11], and decreased alcohol and tobacco consumption [5].

Spillover evidence has also been found in associations between changes in diet and exercise and improvements in psychological variables, such as self-image, anxiety, coping, self-efficacy, and health-related quality of life [6,12]. Likewise, interventions lasting up to two years that have focused on motivational variables (i.e., self-regulation, autonomy support) showed that there was an increase in exercise, fruit and vegetable consumption [13], and self-regulation of eating during weight control [4], which would confirm the spillover effect between these variables.

One possible explanation for the spillover effect is its relationship with confidence and competence in performing healthy behaviors and the transfer of this confidence to other behaviors, either simultaneously (co-occurrence) or sequentially (carry-over), avoiding compensation between behaviors [1,2,13], i.e., balancing an unhealthy behavior with a healthy one, for example, by consuming an excess of sugary foods and compensating by doing more physical exercise.

To understand the association between different healthy behaviors and measure possible transfer effects, Nigg et al. [14] proposed using the transtheoretical model of change by Prochaska and DiClemente [15] as a holistic way of understanding the behavior change process. According to this model, behavior change occurs in a series of five stages: precontemplation, contemplation, preparation, action, and maintenance [15,16].

In the precontemplation stage, people do not consider that they have a problem. In the contemplation stage, people know they have a problem and think seriously about dealing with it through a decisional balance (positive and/or negative implications). In the preparation stage, the person begins to make small changes with the intention of changing and solving the problem. In the action stage, people have already committed to change the behavior and carry it out with a duration of one day to six months. Finally, in the maintenance stage, people avoid relapse and consolidate the behavior by stabilizing it for more than six months. Each of the stages will have cognitive and behavioral challenges that will impact whether the change process consolidates, stabilizes, or relapses. Dutton et al. [17], using the transtheoretical model of change, found evidence that participants in the more advanced stages of change (i.e., action and maintenance) for physical activity reported a significantly higher intake of fruits and vegetables and a reduction in high-fat foods. In general, the application of the transtheoretical model of change through its five stages has been associated with improvements in the body mass index and calorie intake, as well as positive dietary changes, among others [18,19,20].

The transtheoretical model of change and the spillover effect have been associated with other variables, such as motivational types, which have been explained by self-determination theory (SDT) [21]. SDT is a macro theory of motivation that has contributed considerably to predicting numerous health-related behaviors, suggesting that the quality of individuals’ motivation affects the extent to which they engage and persist in behaviors (e.g., exercise or healthy diet). The SDT distinguishes between different types of motivation [22]: autonomous, controlled, and amotivation. Autonomous motivation describes people who become involved in an activity because it is interesting or enjoyable. Controlled motivation refers to behaviors that are performed to satisfy external demands or to receive rewards. Finally, amotivation or absence of regulation describes people with no intention of acting. Authors such as Zamarripa et al. [23] found that amotivated and control-motivated individuals predominated in the first stages of change for physical activity (i.e., precontemplation and contemplation), and these types of motivation decreased in the last stages (i.e., action and maintenance). Thus, autonomous-motivated individuals predominated in the last stages (i.e., action and maintenance). In general, the more autonomous forms of motivation have been more strongly associated with health-related behaviors [24].

The research conducted has been so diverse that it seems essential to establish the most effective pathway towards the adoption of healthy behaviors, particularly physical activity and healthy behaviors for weight control, as well as clarifying the dynamics of types of motivation. In this regard, Nigg et al. [14] proposed weight management as a priority behavior in delivering health interventions, and interventions focused on diet have been compared with psychological approaches (taking into account variables such as autonomy support and self-monitoring), finding that interventions focused on weight control (healthy diet and physical activity) may lead to better results in the attempt to promote more leisure-time physical activity [7,25].

Furthermore, gender and age differences have to be considered when designing the most effective interventions [26]. In relation to physical activity and weight control behaviors, some research has consistently reported that men engage in more physical activity than women, whereas women, compared to men, are more likely to use healthier weight control behaviors, such as low-fat diets, and participate more actively in weight control programs [19,27,28]. Moreover, in the case of weight control, men tend to be more distributed in the precontemplation stage, and women in the maintenance stage [19], but when it comes to physical activity and/or physical exercise, men are mostly located in the maintenance stage and women in the contemplation stage [28,29]. However, Sorensen et al. [29] found that most people, regardless of gender, were in the preparation stage, describing themselves as active, but not regularly. These authors also found that the percentage of people in the precontemplation and maintenance stages increased with age, with relatively more contemplators in younger groups of men and women. These results suggest that physical activity interventions should also be tailored to different age and gender groups [26,29], and so studies need to be conducted to account for possible gender and age differences.

On the basis of the literature reviewed and the theory of the transtheoretical model of change, and with the aim of descriptively showing the spillover effect between physical activity, healthy weight control behaviors, and their motivational correlates, the present study has a twofold objective: (1) to evaluate the association between the study variables (i.e., overall amount of leisure-time physical activity, use of healthy and unhealthy weight control behaviors, and motivational types), and the existence of gender differences in these relationships; (2) to assess the variability in the stage of change for weight control on the basis of the overall amount of leisure-time physical activity, the use of healthy and unhealthy weight control behaviors, and the motivational types, controlling for participants’ age.

## 2. Materials and Methods

### 2.1. Participants

Participants were stratified by gender and age with neighborhood (called “colonias”) as the cluster variable. Of the 912 colonias in Monterrey, 50 were randomly selected. To obtain representativeness, the sample size can be estimated to obtain valid results with a margin of sampling error of ±3% and a confidence level of 95.5%. According to the aforementioned characteristics, it was estimated that the minimum sample to obtain representativeness of the population was 1067 participants, so our data are representative of the city of Monterrey.

The participants were 1219 adults (599 men, 620 women) from Monterrey, Nuevo León, México, from 18 to 65 years old (*M* = 29.37, *SD* = 11.34). By age group, 764 were 18–24 years old, 275 were 30–44 years old, 124 were 45–54 years old, and 56 were 55–65 years old. Regarding the education level, 708 were college graduates, 317 were high school graduates, and 127 were middle school graduates. Furthermore, 716 had a middle-class income, 216 had an upper-middle-class income, and 210 had a lower-middle-class income. All people (100%) contacted agreed to participate in the study.

### 2.2. Measures

The following instruments were administered:

Physical activity was measured with the Godin–Shepard Leisure-Time Physical Activity Questionnaire [30] adapted to Spanish spoken in Mexico. The questionnaire allows for the assessment of self-reported leisure-time physical activity with three intensities (mild, moderate, and strenuous). The participants answered the sentence: “During a typical 7-day period (a week), how many times on the average do you do the following kinds of exercise for more than 15 min during your free time?” Next, participants indicated how many times per week they performed mild-intensity activities (e.g., gentle yoga, gentle walking), moderate-intensity activities (e.g., brisk walking, gentle cycling), and strenuous-intensity activities (e.g., running, vigorous swimming), on a scale from 1 (once a week) to 7 (seven times per week). The score is expressed in units and can be computed in two steps. First, weekly frequencies of mild, moderate, and strenuous activities are multiplied by three, five, and nine, respectively; these three latter values correspond to the metabolic equivalent intensity levels (MET) of the activities listed in the questionnaire. Then, the total weekly leisure activity score is computed in arbitrary units by summing the products of the separate components. The range of scores obtained can vary between 0 and 119 points, indicating the overall amount of physical activity the person does in their free time.

The Stage of Change Questionnaire (URICA-Short form) [31] adapted to weight control and to Spanish spoken in Mexico was used. This scale classifies the individual in a stage of change (precontemplation, contemplation, preparation, action, and maintenance) for weight control. The participants answered the following question: “Have you tried to lose/control your weight?”, marking the option that fits their behavior: (1) Yes, I have done it for more than 6 months; (2) Yes, I have done it for less than 6 months; (3) No, but I will try in the next 30 days; (4) No, but I will try in the next 6 months; and (5) No, and I do not intend to try in the next 6 months.

The Weight-Related Behaviors Scale [32] adapted to Spanish spoken in Mexico was used. It contained 12 items divided into two subscales: Healthy behaviors (e.g., Exercised) with four items, and unhealthy behaviors (e.g., Skipped meals) with eight items. The responses were given on a 5-point Likert type scale from 1 (Never) to 5 (Always), and the question that preceded the items was “During the last four weeks, have you realized any of the following actions for weight loss or to avoid gaining weight?” The goodness of fit indices obtained for the two-factor confirmatory factor analysis (CFA) model in this study were adequate: χ^2^(50) = 381.410; CFI = 0.941; TLI = 0.922; RMSEA = 0.070; SRMR = 0.058.

The Behavioral Regulation in Exercise Questionnaire (BREQ-3) [7] adapted to weight control and to Spanish spoken in Mexico [23] was used. The questionnaire begins with the stem “I control my weight…” and contains 23 items divided into six subscales with four items each, except for identified regulation, which has three items: intrinsic regulation (e.g., because I enjoy the moments when I control my weight), integrated regulation (e.g., because it is consistent with my life goals), identified regulation (e.g., because I value the benefits of controlling my weight), introjected regulation (e.g., because I feel guilty when I don’t), external regulation (e.g., because other people say I should), and amotivation (e.g., I don’t see why I should have to control my weight). Responses were given on a Likert-type scale (0 = definitely not true, 4 = definitely true). The goodness of fit indices obtained in this study testing the six-factor model were adequate: χ^2^(214) = 1685.379; CFI = 0.952; TLI = 0.944; RMSEA = 0.076; SRMR = 0.071. Consistent with the SDT website, items from the intrinsic motivation, integrated, and identified regulation scales were combined to create the autonomous motivation variable, and items from the introjected and external regulation scales were combined to create the controlled motivation variable.

### 2.3. Procedure

We used a non-experimental, quantitative, and cross-sectional design. The study was conducted in accordance with international ethical guidelines consistent with the American Psychological Association and the Institutional Review Board of the University of Nuevo León (México) (REPRIN-FOD-83). The scales were hand-delivered directly to the participants’ homes, where they were informed about the purpose of the study and that they could refuse to answer or withdrawn from the study at any time. They responded in a self-administered manner, freely and anonymously, with the supervision of interviewers, which took 15 to 20 min. Written informed consent was obtained prior to the data collection.

### 2.4. Data Analysis

Before analyzing the data, the dataset was screened for multivariate outliers, and the normality of the distributions was also examined. Missing data were very small (<0.1%), and so we decided not to replace these scores. When less than 5% of the data are missing in the random dataset, it is not likely to be a serious problem.

Descriptive statistics, Pearson’s correlations, and Cronbach’s alpha of all the study variables were calculated. A *t*-test for independent samples was used to find out if there were gender differences in the study variables. Fisher’s *z*-transformation test was used to examine whether the associations between the variables were similar in both genders. Chi-squared tests were used to test for differences in the distribution of the participants in the different stages of change for weight control by gender, and effect sizes were estimated with Cramer’s V. The differences in the means of the study variables across the stages of change were analyzed using multivariate analysis of variance (MANCOVA), controlling for age, and post hoc Tukey or Games Howell tests. To run the MANCOVA analysis, we took into account the assumptions of normality (skewness and kurtosis), homogeneity of variances (Levene’s test), and homogeneity of covariances (Box’s test of equality of covariance matrices). For these analyses, we used the IBM SPSS Statistics version 25.

## 3. Results

Participants’ responses showed that values for healthy weight control behaviors, as well as autonomous motivation, were above the mean value of the instruments, whereas global physical activity, unhealthy weight control behavior, controlled motivation, and amotivation values were below the mean value. Correlation analysis showed that all the variables correlated significantly with each other in the expected direction, with some exceptions that were non-significant (see Table 1).

Table 2 shows that men scored higher on global physical activity, unhealthy weight control behaviors, and amotivation than women. Women scored higher on healthy weight control behaviors than men. Results of the gender differences in the correlations showed that there were no significant differences in any of the studied variables (see Table 3).

Table 4 shows that most of the participants were in the maintenance stage (26.9%), followed by the action (23.6%), contemplation (20.3%), precontemplation (14.8%), and preparation stages (14.4%). Males and females are placed in all the stages of change with similar frequency (χ^2^(2) = 6.89, *p* = 0.14), with a low association between gender and stages of change for weight control (Cramer’s V = 0.08).

The results of the MANCOVA revealed statistically significant differences between the different stages of change for all the study variables, controlling for age (Λ Wilks = 0.671; *F* = 15.62; df = 32; *p* = 0.01; η^2^ = 0.10) (see Table 5). As can be seen in Table 5, eta squared was low for unhealthy weight control behavior and for amotivation. It was medium for physical activity and controlled motivation, and eta squared was high for healthy weight control behavior and for autonomous motivation (precisely the two variables considered to be the healthiest).

**Figure 1:**
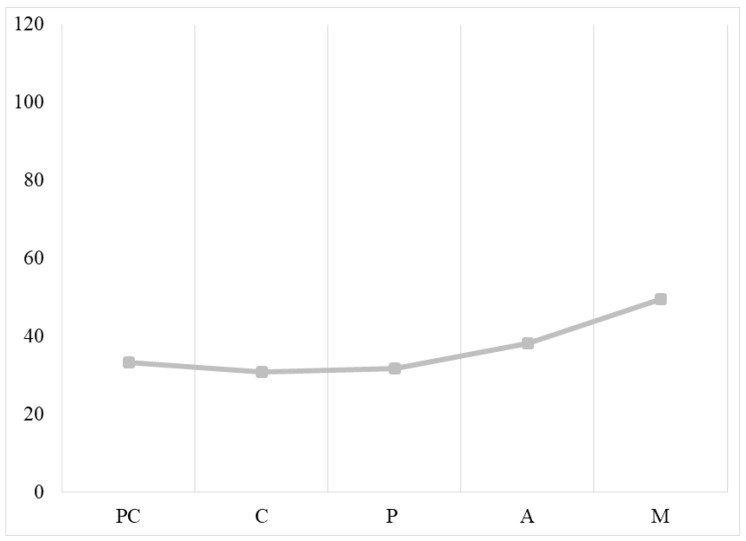
Differences in global physical activity across the stages of change for weight control. Note: PC = precontemplation, C = contemplation, P = preparation, AC = action, M = maintenance.

Regarding physical activity, post hoc tests showed that people in the maintenance stage had a higher level of global physical activity than people in the other stages of change, but there were no significant differences between people in the precontemplation and action stages. Moreover, there were no significant differences between people in the precontemplation, contemplation, and preparation stages (see Figure 1).

**Figure 2:**
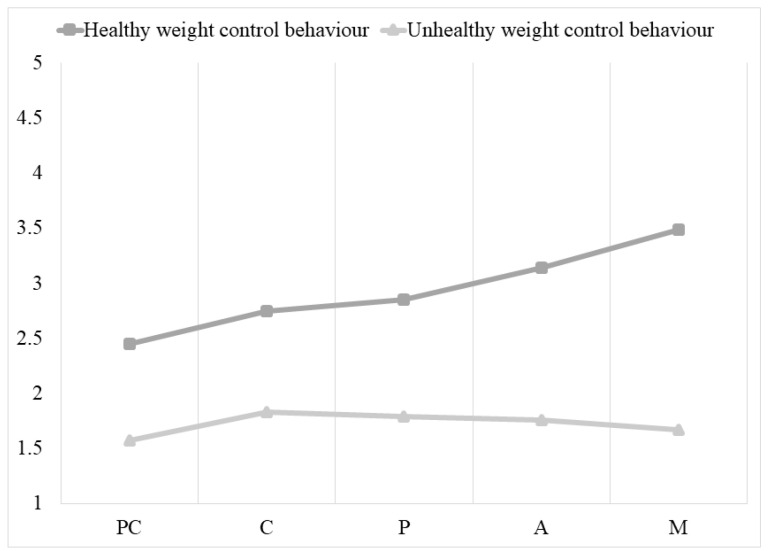
Differences in healthy and unhealthy weight control behavior across the stages of change for weight control. Note: PC = precontemplation, C = contemplation, P = preparation, AC = action, M = maintenance.

For healthy weight control behavior, participants in the maintenance stage performed healthier weight control behaviors than people in the other stages of change, but there were no significant differences in healthy weight control behavior between people in the contemplation and preparation stages. For unhealthy weight control behavior, post hoc tests only revealed significant differences between the precontemplation and contemplation stages (with higher mean values for unhealthy weight control behaviors in the contemplation stage), and no other differences were found between the other stages of change (see Figure 2).

**Figure 3:**
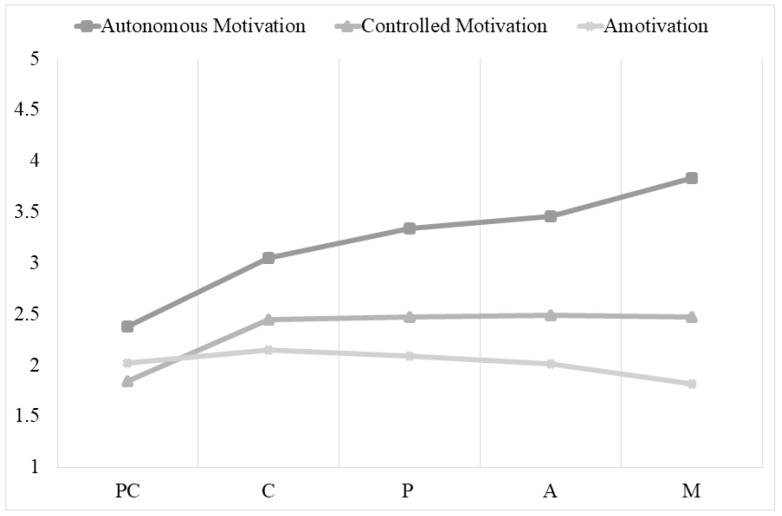
Differences in types of motivation across the stages of change for weight control. Note: PC = precontemplation, C = contemplation, P = preparation, AC = action, M = maintenance.

Finally, regarding autonomous motivation, participants in the maintenance stage had significantly higher autonomous motivation than participants in any of the other stages. There were no differences between the preparation and action stages. With regard to controlled motivation, participants in precontemplation had significantly lower controlled motivation than participants in the other stages, and there were no significant differences between participants in the contemplation, preparation, action, and maintenance stages. Regarding amotivation, people in the maintenance stage had significantly less amotivation than people in the other stages of change; however, individuals in the maintenance stage did not differ significantly from individuals in the action stage. In addition, no differences were found between individuals in the precontemplation, contemplation, preparation, and action stages (see Figure 3).

## 4. Discussion

The spillover effect points to the association between healthy behaviors and between unhealthy behaviors, and as well as an interaction between the two [1,2], such that engaging in a healthy behavior leads to an increase in other healthy behaviors and a reduction in unhealthy behaviors. In line with these suggestions, on the one hand, the results of this study showed positive associations between healthy behaviors (physical activity and healthy weight control behaviors) and positive associations between healthy behaviors and adaptive motivational variable (i.e., autonomous motivation). On the other hand, we found positive associations between unhealthy behaviors (i.e., unhealthy weight control behaviors) and maladaptive motivational variables (i.e., controlled motivation and amotivation). Finally, the results showed negative relationships between healthy behaviors and lower quality motivation type (i.e., between global physical activity and healthy weight control behaviors and amotivation) (see Table 1).

With regard to gender differences, our results revealed that men engage in more physical activity and unhealthier weight control behaviors than women, whereas women engage more in healthy weight control behaviors (see Table 2) [19,27,28]. However, in the distribution of stages of change for weight control, we did not find differences associated with gender (see Table 4), in contrast to other authors’ results [19,28,29].

Several previous studies tried to test the spillover effect in interventions, and although the results were certainly inconsistent, there is agreement about the close relationship between healthy behaviors and their importance in eliciting consequential health effects [3,33].

The results of the present study point to the validity of the transtheoretical model of change for assessing behavioral change in health, allowing us to visualize the association between weight control behavior, overall physical activity, healthy and unhealthy weight control behaviors, and motivational variables (see Table 5) [3]. When focusing on physical activity, we observed that participants in the maintenance stage engage in more physical activity than those in earlier stages. These results show that people who have been managing their weight for more than six months also engage in more frequent and/or intense physical activity (see Figure 1).

Focusing on healthy weight control behaviors, we observed that they gradually increase until their use becomes significant in the action stage and during maintenance (see Figure 2). These results indicate that only people who are committed to change will significantly implement healthy ways of controlling their weight (e.g., decreasing products high in fat and sugar, increasing consumption of fruit and vegetables). These findings agree with what was proposed by Baker and Brownell [3] and confirmed in subsequent research [5,6,11]. In contrast, unhealthy weight control behaviors increase significantly in the contemplation stage and then gradually decrease, without disappearing in the maintenance stage.

Interestingly, when comparing overall physical activity and healthy weight control behaviors, the latter increase significantly earlier than the former. Therefore, people who commit to change and take action may perceive themselves as more competent in increasing their intake of fruit and vegetables and decreasing high fat and high sugar foods, etc., than engaging in physical activity behaviors. This perception may have an impact on their confidence and help them to engage more effectively in change through healthy weight control behaviors [1,2,13].

Focusing on motivational variables, autonomous motivation increases in the maintenance stage, implying that individuals who are moving from one stage to the next increasingly strengthen their internal reasons for weight control. At the same time, controlled motivation and amotivation decrease from the contemplation stage onwards (see Figure 3). These results are similar to those of Mata et al. [4] and Zamarripa et al. [23], who found a higher level of autonomy as change improved and in the later stages of change (i.e., action and maintenance).

In general, the stages of change have been described conceptually, but without elaborating on each of them. This study highlights the importance of the contemplation stage and the maintenance stage, where significant differences were observed (compared to the other stages of change) in all the variables studied. The contemplation stage was found to be a crucial stage for change that can destabilize the behavior the person currently carries out. This stage is generally perceived as a passive or non-active stage of change [15,16]. However, we can argue that the contemplation stage becomes an empowering stage of change because, if it successfully leads into the preparation stage, the person will be much more likely to continue to move through the stages of change. Likewise, the present study, on the whole, points out the stages of greatest risk in weight control strategies, as well as the pre-contemplative and, above all, contemplative stages. Once the person starts to perform the behavior, weight control strategies become healthier, probably due to the differentiation of the stages. It is more difficult to maintain unhealthy weight control behavior over time than healthy weight control strategies.

Thus, it is important to change and commit to change, but it is much more important to be able to reach the maintenance stage because, during this stage, substantial changes are achieved and consolidated, thus representing adherence to physical activity and weight control behavior [15,16] and the transfer between healthy behaviors or the spillover effect [4,5].

## 5. Limitations

The present study has several limitations. First, the use of a cross-sectional design does not allow us to state cause–effect relationships among the study variables. Second, the sample used in the study was composed of participants from only one municipality, which limits the generalizability of our results. Third, data were collected using self-report instruments, which could have been influenced by social desirability. Therefore, future studies should replicate the findings reported here using objective measures. These limitations notwithstanding, the present study is a good starting point for future longitudinal and experimental research, with the aim of delving into the spillover phenomenon.

## 6. Practical Implications

Practical implications of the study have to do with using the transtheoretical model of change as a starting point for assessing change, intervening in the basis of weight control and training people in the appropriate way to engage in more physical activity in the leisure time. In addition, follow-ups during the intervention will be crucial in order to accompany the person towards the maintenance stage. This idea is in line with the one proposed by Powell et al. [34], who showed that the follow-up of participants in an intervention is crucial for the continuity of weight loss, given that it is associated with maintaining changes, the spillover phenomenon, and avoiding relapses [4,5]. In this follow-up, an approach based on autonomy support is recommended [23,35] in order to enhance the connection between physical activity; weight control behaviors; and the interests, values, and beliefs of the individuals in this process and focus on healthy strategies. In sum, educational, health, and political authorities need to ensure that healthy community strategies are being applied continuously.

## 7. Conclusions

This study points to the close relationship between physical activity, healthy behaviors, and their association with motivational variables [4,6,13]. Furthermore, the transtheoretical model of change seems to be a good starting point for understanding the dynamics between physical activity and healthy behaviors [14], again highlighting the importance of contemplation and maintenance stages in healthy change. The study highlights that healthy weight control behavior, which includes diet management, is a behavior over which individuals are likely to perceive themselves as more competent and may lead them to increase the amount of leisure-time physical activity, generating more healthy behaviors and, thus, the spillover effect.

## Figures and Tables

**Table 1:** Descriptive statistics, correlations, and reliabilities for study variables (*n* = 1219).

Variables	*M*	*SD*	Alpha	1	2	3	4	5
Global physical activity	37.87	25.91		-				
2. Healthy weight control behavior	3.01	0.90	0.72	0.41 **	-			
3. Unhealthy weight control behavior	1.73	0.75	0.84	0.06 *	0.05	-		
4. Autonomous motivation	3.30	1.01	0.92	0.25 **	0.44 **	−0.08	-	
5. Controlled motivation	2.38	0.84	0.83	0.05	0.12 **	0.42 **	0.37 **	-
6. Amotivation	2.02	0.97	0.74	−0.07 *	−0.14 **	0.43 **	−0.13 **	0.46 **

Note: Range variables: 1–5, except for global physical activity: 0–119. ** *p* < 0.01; * *p* < 0.05.

**Table 2:** Gender differences in the study variables.

Variables	Male (*n* = 599)		Female (*n* = 620)		
	*M*	*SD*	*M*	*SD*	*t*
Global physical activity	40.38	26.50	35.43	25.08	3.35 **
Healthy weight control behaviour	2.96	0.91	3.07	0.90	−2.05 *
Unhealthy weight control behaviour	1.78	0.80	1.68	0.70	2.30 *
Autonomous motivation	3.25	1.04	3.34	0.98	−1.53
Controlled motivation	2.38	0.82	2.38	0.85	−0.01
Amotivation	2.16	0.98	1.88	0.93	5.09 **

Note: Range variables: 1–5, except for global physical activity: 0–119. ** *p* < 0.01; * *p* < 0.05.

**Table 3:** Results of values of correlation differences by gender for the study variables.

Variables Correlated	Male (*n* = 599) Correlation	Female (*n* = 620) Correlation	*z*
Physical activity–Healthy WCB	0.44 **	0.40 *	0.74
Physical activity–Unhealthy WCB	0.05	0.06	−0.21
Physical activity–Autonomous motivation	0.27 **	0.24 **	0.56
Physical activity–Controlled motivation	0.05	0.04	0.21
Physical activity–Amotivation	−0.01	−0.07	−0.42
Healthy WCB–Unhealthy WCB	0.08 *	0.01	1.27
Healthy WCB–Autonomous motivation	0.43 **	0.46 **	−0.73
Healthy WCB–Controlled motivation	0.16 **	0.10*	1.07
Healthy WCB–Amotivation	−0.10 *	−0.17 **	1.17
Unhealthy WCB–Autonomous motivation	0.01	−0.01	0.21
Unhealthy WCB–Controlled motivation	0.42 **	0.42 **	−0.02
Unhealthy WCB–Amotivation	−0.43 **	−0.43 **	−0.13
Autonomous motivation–Controlled motivation	0.40 **	0.33 **	0.15
Autonomous motivation–Amotivation	−0.12 **	−0.12 **	0.00
Controlled motivation–Amotivation	0.47 **	0.46 **	0.33

Note: ** *p* < 0.01, * *p* < 0.05. WCB = weight control behavior.

**Table 4:** Distribution of the categorization of the sample in stages of change (*n* = 1219).

*Have You Tried to Lose/Control Your Weight?*	Stage	*f*	%	Male %	Female %
Yes, I have done it for more than 6 months	Maintenance	328	26.9	27.7	26.1
Yes, I have done it for less than 6 months	Action	288	23.6	21.4	25.8
No, but I will try in the next 30 days	Preparation	175	14.4	13.0	15.2
No, but I will try in the next 6 months	Contemplation	247	20.3	21.9	18.7
No, and I do not intend to try in the next 6 months	Precontemplation	181	14.8	16.0	13.7
	*Total*	1219	100		

Note: *f* = frequency; % = percentage.

**Table 5:** MANCOVA testing differences between stages of change for the study variables.

Variables	*F*	df	*p*	*Eta* ^2^	*Levene*	*p*
Physical activity	27.48	4	0.01	0.08	2.45	0.05
Healthy weight control behaviour	56.21	4	0.01	0.16	5.26	0.01
Unhealthy weight control behaviour	4.19	4	0.02	0.01	1.30	0.27
Autonomous motivation	84.50	4	0.01	0.22	5.49	0.01
Controlled motivation	23.15	4	0.01	0.07	0.32	0.86
Amotivation	5.67	4	0.01	0.02	0.16	0.96

## Data Availability

Not applicable.
